# Depression, anxiety and stress, during COVID-19 pandemic among midwives in ethiopia: A nationwide cross-sectional survey

**DOI:** 10.3389/fpsyt.2022.867040

**Published:** 2022-07-26

**Authors:** Eskeziaw Abebe Kassahun, Belayneh Ayanaw Kassie, Sewbesew Yitayih Tilahun, Asmamaw Demis Bizuneh

**Affiliations:** ^1^School of Midwifery, College of Health Sciences, Woldia University, Woldia, Ethiopia; ^2^Department of Women’s and Family Health, School of Midwifery, College of Medicine and Health Sciences, University of Gondar, Gondar, Ethiopia; ^3^Department of Psychiatry, College of Medicine and Health Sciences, University of Gondar, Gondar, Ethiopia; ^4^School of Nursing, College of Health Sciences, Woldia University, Woldia, Ethiopia

**Keywords:** anxiety, depression, stress, COVID-19, midwives, Ethiopia

## Abstract

**Introduction:**

COVID-19 has rapidly crossed borders, infecting people throughout the whole world, and has led to a wide range of psychological sequelae. Midwives who come close in contact with women while providing care are often left stricken with inadequate protection from contamination with COVID-19. Therefore, this study aimed to assess the level of depression, anxiety, and stress (DASS) among midwives in Ethiopia.

**Methods:**

A cross-sectional study was conducted from 20 June to 20 August 2020, among 1,691 practicing midwives in Ethiopia. A simple random sampling technique was used to select study participants. Data were collected through a structured telephone interview. A 21-item depression, anxiety, and stress scale (DASS-21) was used. Data were entered using the Google forms platform and were analyzed with SPSS version 24. Both bivariate and multivariable logistic regression analyses were employed. Variables with a *p-*value < 0.05 in the final model were declared statistically significant. Adjusted odds ratio (*AOR*) with the corresponding 95% confidence interval (95% *CI*) was used to determine independent predictors.

**Results:**

The prevalence of DASS among midwives in Ethiopia was 41.1, 29.6, and 19.0%, respectively. Being female [*AOR* = 1.35; 95% *CI*: 1.08, 1.69], working in rural areas [*AOR* = 1.39; 95% *CI*: 1.06, 1.82], having poor knowledge of COVID-19 [*AOR* = 1.40; 95% *CI*: 1.12, 1.75], having poor preventive practice [*AOR* = 1.83; 95% *CI*: 1.47, 2.28], and substance use [*AOR* = 0.31; 95% *CI*: 0.17, 0.56] were significantly associated with depression; while, working in the governmental health facility [*AOR* = 2.44; 95% *CI*: 1.24, 4.78], having poor preventive practice [*AOR* = 1,47; 95% *CI*: 1.16, 1.85], and having poor attitude [*AOR* = 2.22; 95% *CI*: 1.04, 1.66] were significantly associated with anxiety. Furthermore, working in rural areas [*AOR* = 0.57; 95% *CI*: 0.39, 0.83], substance use [*AOR* = 2.06; 95% *CI*: 1.51, 2.81], having poor knowledge [*AOR* = 1.44; 95% *CI*: 1.20, 1.90], and having poor preventive practice [*AOR* = 1.60; 95% *CI*: 1.23, 2.10] were associated with stress.

**Conclusion:**

In this study, the overall magnitude of depression, anxiety, and stress were high. Addressing knowledge gaps through information, training, and safety protocols on COVID-19 and the provision of adequate personal protective equipment (PPE) is essential to preserve the mental health of Midwives during COVID-19.

## Introduction

COVID-19 is an infectious disease that is caused by a new strain of novel coronavirus and is known to cause illnesses ranging from the common cold to severe acute respiratory syndrome (SARS). It was first confirmed in December 2019 in Wuhan, Hubei Province, China (1). In January 2020, the World Health Organization (WHO) declared that COVID-19 is a Public Health Emergency of International Concern (2). Patients with COVID-19 either have an asymptomatic disease or are present with symptoms, such as fever, cough, or shortness of breath (3).

COVID-19 has rapidly crossed borders, infecting people throughout the whole world. Its emergence and spread cause confusion, anxiety, and fear among the general public (4). As research into COVID-19 continues, a lot of the facts keep on changing and many myths are also prevalent in the general population regarding the prevention and management of the infection. In the time of widespread use of social media, these myths along with fake news about corona are also spreading rapidly, which causes feelings of stress, anxiety, and depression (5).

Depression, anxiety, and stress (DASS) are globally affecting every individual to variable extents. Recent evidence suggests that individuals who are kept in isolation and quarantine experience significant distress in the form of anxiety, anger, confusion, and post-traumatic stress symptoms (6). With this mode of transmission, healthcare workers are among the highest risk of being infected. The highly contagious COVID-19 virus is an additional hazard for the healthcare system apart from the burden of extended work hours, physical and psychological stress, burnout, and fatigue. Midwives are essential in reducing maternal and perinatal morbidity and mortality but are an all-too-often forgotten human resource in healthcare systems (7, 8).

Due to their direct contact with patients, healthcare workers are among the highest risk of being infected by COVID-19 (9) and suffering psychological exhaustion (10) after working in an extremely stressful environment. The United Nations (UN) body reported that more than 1.4 million infections of COVID-19 in the world are accounted for by health workers, at least 10% of all cases (11). In Africa, the information on health worker infections is still limited, but preliminary data reported that over 10,000 health workers in Africa were infected with COVID-19 (12).

In Ethiopia, during the emergence of the pandemic outbreak, healthcare services were partially closed and focused on the prevention and treatment of COVID-19. However, midwifery services were among the services that continued as usual. Midwives, who come close and prolonged contact with women when providing care, are often left stricken with inadequate protections from contamination, high risks of infection, working burnout, fear, anxiety, and depression. Furthermore, the limited availability of adequate personal protective equipment (PPE) has raised concerns and worries about the risk of COVID-19 infection. However, there is no known information on the psychological impact of COVID-19 among midwives. Therefore, this study aimed to assess the prevalence of DASS during the COVID-19 pandemic among midwives in Ethiopia in 2020.

## Materials and methods

### Study setting, design, and period

A web-based quantitative cross-sectional study was conducted among midwives who are providing maternal health services in Ethiopia from 20 May to 20 August 2020. According to the Ethiopian Midwives Association (EMA) database in 2020, there are 16,925 midwives in Ethiopia.

### Source and study population

All midwives currently providing clinical care in Ethiopia were the source population, and randomly selected midwives were the study population. All midwives providing clinical care for reproductive, maternal, neonatal, child, and adolescent health were included.

### Sample size determination and sampling procedure

The sample size was determined using the single population proportion formula, taking the following assumptions: 20.1% depression symptoms in China due to COVID-19 outbreak (13), 95% confidence interval (*CI*), *Z* as 1.96, 2% margin of error, and adding 10% non-response rate. Finally, 1,691 was the final sample size considered in the current study.

The total sample was proportionally allocated for all administrative regional states and two city administrations based on their number of midwives according to the EMA national census database. Then, a simple random sampling technique was used to select study participants. Then, data were collected through a telephone interview and each response was entered in the prepared Google forms platform data collection tool.

### Data collection tools and measurement

Due to the COVID-19 contagious pandemic outbreak, the team opted to collect the data through telephone interviews. A pretested and structured telephone interviewer-administered questionnaire was used to collect the data. For those study participants who did not pick up their phones, whose phones were not working, and who were not ready to talk by the time, and then our data collectors called them on another day during the data collection period.

The questionnaire consisted of three parts, such as socio-demographic characteristics, COVID-19-related knowledge, attitude, and practice, and depression, anxiety, and stress scale (DASS-21). Depression, anxiety, and stress have 7 scales, with a 4-point Likert scale from 0 to 3, where 0 stands for “never–did not apply to me at all,” 1 stands for “sometimes–applied to me to some degree, or some of the times,” 2 stands for “often, i.e., applied to me to a considerable degree or a good part of the time,” and 3 stands for “almost always applied to me very much or most of the time,” and the final score of each part was obtained by adding the scores of the related questions and finally multiplied by 2, thus arriving at three separate scores for all three subscales (14).

#### Depression, anxiety, and stress

A respondent who scored below 10, 8, and 15 for each of the respective questions on the DASS-21 scale was considered as having no DASS and a respondent who scored ≥ 10, ≥ 8, and ≥ 15 was considered as having DASS, respectively (14).

#### Good knowledge of COVID-19

A respondent who answered the mean and above score (9.91) among sixteen item knowledge questions was considered as having “good knowledge” of COVID-19.

#### Favorable attitude toward COVID-19

A respondent who answered the mean and above score (3.25) among seven-item attitude questions was considered as having a “favorable attitude” toward COVID-19.

#### Good COVID-19 prevention practices

A respondent who answered the mean and above score (3.62) among five-item prevention practice questions was considered as having “good preventive practice “on COVID-19 prevention.

The questionnaire was pretested among midwives working in academic institutions to ensure the validity of the tool, and then the correction was made before the actual data collection started. The training was given to data collectors and supervisors on the data collection procedures. In total, fifteen multilingual BSc nurses collected the data through a telephone interview and the data were supervised by two masters of public health holders. Proper coding and categorization of data were maintained for the quality of the data to be analyzed. Double data entry was done and any inconsistency was managed accordingly.

### Data analysis procedure

The collected data were extracted from Google forms and exported to Microsoft Excel for cleaning and coding. The data were analyzed using SPSS version 24 software (IBM Corp., Released 2016. IBM SPSS Statistics for Windows, Version 24.0. Armonk, NY: IBM Corp.). Frequencies and cross-tabulations were used to summarize descriptive statistics of the data. Tables and graphs were used for data presentation. Binary logistic regression analysis was primarily used to establish an association between dependent and independent variables. Then, variables with a *p*-value of less than 0.2 fitted into multiple logistic regression models and variables having a *p*-value less than 0.05 in the multivariable logistic regression analysis were considered to declare the statistical significance. The strength of association was interpreted using the adjusted odds ratio (*OR*) and 95% *CI*.

### Ethics statement

The study was approved by the Institutional Review Boards of Woldia University (Ref: WDU/986/RCS/2020). Informed consent was obtained from participants after providing all the necessary information on the study, informing them of the purpose, benefit, risk, and confidentiality of the information and the voluntary nature of the participation in the study. Participants were informed that all data obtained from them were kept confidential and no identifying information was collected from them.

## Results

### Socio-demographic characteristics

A total of 1,498 participants were included in the study, which makes a response rate of 88.6%. The study participants with symptoms and signs of COVID-19 were referred to nearby isolation centers. The median age of the participants was 27.0 (± 4.3) years. The majority (93.3%) of participants were in the age range of 16–34 years, 781 (52.1%) were men and 769 (51.3%) were married. Moreover, more than four-fifths (81.4%) are working in an urban area. The mean (± SD) work experience of participants was 5.72 (± 4.1) years with nearly three-fifths (58.3%) having less than 5 years of work experience (Table 1).

**TABLE 1:** Socio-demographic characteristics of midwives working in Ethiopia in 2020 (*n* = 1,498).

Variables	Category	Frequency (*n*)	Percent (%)
Age	16–34	1,398	93.3
	35–62	100	6.7
Sex	Male	781	52.1
	Female	717	47.9
Marital status	Married	769	51.3
	Single	729	48.7
Resident	Urban	1,219	81.4
	Rural	279	18.6
Religious status	Orthodox	964	64.3
	Muslim	308	20.6
	Protestant	210	14.0
	Others*	16	1.1
Educational level	Diploma	539	36.0
	Bachelor degree	837	55.9
	Master’s degree	122	8.1
Work experience	≤5 years	874	58.3
	5–10 years	506	33.8
	>10 years	118	7.9
Type of health facility	Governmental	1,427	95.3
	Private	71	4.7
Health facility (*n* = 1,427)	Hospital	778	54.5
	Health center	649	45.5

Others*, Apostolic, Catholic, Adventist 7th day.

### Knowledge, attitude, and preventive practices toward COVID-19

In this study, 876 (58.5%) midwives had good knowledge of COVID-19. Regarding attitude and preventive practices of COVID-19, 590 (39.4%) of midwives had a favorable attitude and 854 (57.0%) had good preventive practices toward COVID-19.

### Prevalence of depression, anxiety, and stress

**FIGURE 1:**
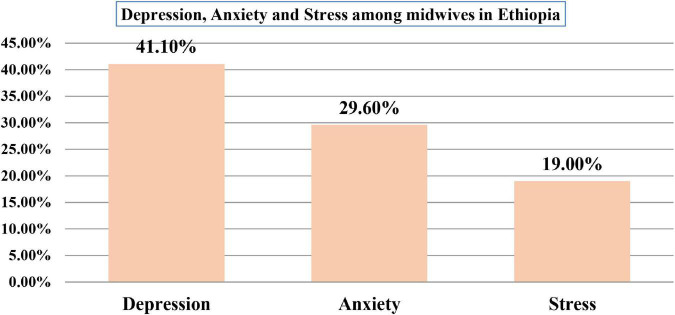
The level of depression, anxiety, and stress among midwives in Ethiopia in 2020.

The overall prevalence of DASS among midwives was found to be 41.1% [95% *CI*: 38.6, 43.7)], 29.6% [95% *CI*: 27.3, 31.8], and 19.0% [95% *CI*: 17.0, 20.8], respectively (Figure 1).

### Factors associated with depression

In the bivariable analysis: age, sex, marital status, residence, educational level, type of working facility, substance use (tobacco, khat, or alcohol), knowledge of COVID-19, attitude toward COVID-19, and preventive practice toward COVID-19 were found to be significantly associated with depression. After controlling confounders through the multivariable analysis, sex, residence, types of working health facility, knowledge, preventive practice on COVID-19 pandemic prevention and substance use were significantly associated with depression among midwives in Ethiopia.

Female midwives were 1.35 times more likely to develop depression as compared with men [adjusted odds ratio *[AOR]* = 1.35; 95% *CI*: 1.08, 1.69]. Those midwives who are working in the rural area were 1.39 times more likely to develop depression as compared with midwives working in the urban areas [*AOR* = 1.39; 95% *CI*: 1.06, 1.82]. The odds of developing depression among midwives with the poor knowledge of COVID-19 were 1.40 times higher compared with their counterparts [*AOR* = 1.40; 95% *CI*: 1.12, 1.75]. Those midwives with a poor COVID-19 prevention practice were 1.83 times more likely to develop depression as compared with their counterparts [*AOR* = 1.83; 95% *CI*: 1.47, 2.28]. The odds of depression were 1.69 times higher among midwives who were exposed to substances than non-users [*AOR* = 1.69; 95% *CI*: 1.27, 2.25]. However, the odds of having depression were decreased by 69% among midwives working in governmental health facilities than midwives working in private health facilities [*AOR* = 0.31; 95% *CI*: 0.17, 0.56] (Table 2).

**TABLE 2:** Bivariable and multivariable logistic regression analysis for factors associated with depression among midwives in Ethiopia in 2020 (*n* = 1,498).

Variables	Category	Depression		COR (95%CI)	AOR (95%CI)
		Yes (%)	No (%)		
Sex	Male	293 (37.5)	488 (62.5)	1	1
	Female	323 (45.0)	394 (55.0)	1.37 (1.11, 1.68)	1.35 (1.08, 1.69) *
Residence	Urban	484 (39.7)	735 (60.3)	1	1
	Rural	132 (47.3)	147 (52.7)	1.36 (1.05, 177)	1.39 (1.06, 1.83)
Working facility	Government	562 (39.4)	865 (60.6)	0.21 (0.12, 0.36)	0.31 (0.17, 0.56) **
	Private	54 (76.1)	17 (23.9)	1	1
Knowledge on COVID-19	Good	315 (36.0)	561 (64.0)	1	1
	Poor	301 (48.4)	321 (51.6)	1.67 (1.35, 2.06)	1.40 (1.12, 1.75) *
COVID-19 prevention practice	Good	288 (33.7)	566 (66.3)	1	1
	Poor	328 (50.9)	316 (49.1)	2.04 (1.65, 2.52)	1.83 (1.47, 2.28) **
Substance use	Yes	142 (53.4)	124 (46.6)	1.83 (1.40, 2.39)	1.69 (1.27, 2.25) **
	No	474 (38.5)	758 (61.5)	1	1

*p < 0.005, **p < 0.001, and 1: constant.

### Factors associated with anxiety

In the bivariable analysis, the type of working facility, COVID-19 preventive practice, and attitude toward COVID-19 were significantly associated with anxiety among midwives. The odds of anxiety were 2.44 times higher among midwives who are working in government health facilities as compared with midwives working in private health facilities [*AOR* = 2.44; 95% *CI*: 1.24, 4.78]. The odds of anxiety were 1.47 times higher among midwives who had a poor COVID-19 preventive practice as compared with their counterparts [*AOR* = 1.47; 95% *CI*: 1.16, 1.85]. Moreover, the odds of anxiety were 1.31 times higher among midwives with an unfavorable attitude toward COVID-19 compared with those with a poor attitude [*AOR* = 1.31; 95% *CI*: 1.04, 1.66] (Table 3).

**TABLE 3:** Bivariable and multivariable logistic regression analysis for factors associated with anxiety among midwives in Ethiopia in 2020 (*n* = 1,498).

Variable	Categories	Anxiety		COR (95% CI)	AOR (95% CI)
		Yes (%)	No (%)		
Educational level	Diploma	142 (26.3)	397 (73.7)	1.25 (0.98, 1.59)	
	BSc degree	258 (30.8)	579 (69.2)	1.52 (1.00, 2.31)	
	MSc degree	43 (35.2)	79 (64.8)	1	1
Marital status	Single	197 (27.4)	523 (72.6)	1	1
	Married	246 (31.6)	532 (68.4)	1.27 (1.02, 1.59)	
Working facility	Government	432 (30.3)	995 (69.7)	2.37 (1.23, 4.55)	2.44 (1.24, 4.78)*
	Private	11 (15.5)	60 (84.5)	1	1
Substance use	Yes	93 (35.0)	173 (65.0)	1.36 (1.02, 1.79)	
	No	350 (28.4)	882 (71.6)	1	1
COVID-19 prevention practice	Poor	218 (33.9)	426 (66.1)	1.43 (1.14, 1.79)	1.47 (1.16, 1.85)*
	Good	255 (28.8)	629 (71.2)	1	1
COVID-19 attitude	Unfavorable	290 (31.9)	618 (68.1)	1.34 (1.10, 1.69)	1.31 (1.04, 1.66)*
	Favorable	153 (25.9)	437 (64.1)	1	1

*p < 0.005, and 1: constant.

### Factors associated with stress

In multivariable logistic regression analysis: residence, substance use, knowledge of midwives toward COVID-19, and COVID-19 pandemic prevention practices were significantly associated with depression among midwives in Ethiopia. Unlike depression, those midwives who are working in rural areas have decreased the odds of stress by 43% as compared with midwives working in the urban area [*AOR* = 0.57; 95% *CI*: 0.39, 0.83]. The odds of stress were 2.06 times more likely among midwives who use substances than their counterparts [*AOR* = 2.06; 95% *CI*: 1.51, 2.81]. The odds of stress were 1.44 times more likely among midwives who had poor knowledge than their counterparts [*AOR* = 1.44; 95% *CI*: 1.20, 1.90]. Those midwives who had poor COVID-19 prevention practice were 1.60 times more likely to develop stress as compared with those who had good preventive practice [*AOR* = 1.60; 95% *CI*:1.23, 2.10] (Table 4).

**TABLE 4:** Bivariable and multivariable logistic regression analysis for factors associated with stress among midwives in Ethiopia in 2020 (*n* = 1,498).

Variables	Categories	Stress		COR (95% CI)	AOR (95% CI)
		Yes (%)	No (%)		
Marital status	Single	128 (17.6)	601 (82.4)	1	1
	Married	156 (20.3)	613 (79.7)	1.20 (0.92, 1.55)	
Residence	Urban	248 (20.3)	971 (79.7)	1	1
	Rural	36 (12.9)	243 (87.1)	0.58 (0.40, 0.85)	0.57 (0.39, 0.83)*
Working facility	Government	258 (18.1)	1,169 (71.9)	1	1
	Private	26 (36.6)	45 (63.4)	2.62 (1.59, 4.32)	
Substance use	Yes	83 (31.2)	183 (69.8)	2.33 (1.72, 3.14)	2.06 (1.51, 2.81)**
	No	201 (16.3)	1,031 (83.7)	1	1
COVID-19 knowledge	Poor	141 (22.7)	481 (77.3)	1.50 (1.16, 1.95)	1.44 (1.20,1.90)*
	Good	143 (16.3)	733 (83.7)	1	1
COVID-19 prevention practice	Poor	158 (24.5)	486 (75.5)	1.88 (1.45, 2.44)	1.60 (1.23, 2.10)*
	Good	126 (14.8)	728 (85.2)	1	1

*p < 0.005, **p < 0.001, and 1: constant.

## Discussion

To the best of our knowledge, this work is the first nationwide survey on the prevalence of DASS among midwives working in clinical practice following the COVID-19 pandemic. The overall prevalence of DASS among midwives in Ethiopia was found to be 41.1, 29.6, and 19.0%, respectively. This finding is lower than another study conducted in Ethiopia; depression (60.3%), anxiety (78%), and stress (33.8%) (15); in northwest Ethiopia, depression (55.3%), anxiety (69.6%), and stress (79.5%) (16); in Turkey, depression (64.7%), anxiety (51.6%), and stress (41.2%) among the health workers (17); in New York, depression (48%), anxiety (33%), and stress (57%) (18), and in China, depression (50.4%), anxiety (44.6%), and stress (71.5%) (19). This might be due to the fact that those studies were done during the early stage of COVID-19, at which the outbreak was severe and caused fear and frustration among the healthcare providers and the general public. In addition, little is known about the virus, including prevention and transmission means, clinical presentation, and personal protective equipment during the initial period of the pandemic.

However, the finding of this study was higher than that of the study conducted in Singapore among healthcare workers, in which 14.5% of participants screened positive for anxiety, 8.9% for depression, and 6.6% for stress (16). The possible reason could be the difference in perceived exposure among healthcare providers; midwives cannot keep their physical distance during antenatal care (ANC), labor, and delivery, because of the nature of services that require close and prolonged contact with women. Additionally, it is difficult to wear a face mask during labor and delivery the whole day, which creates discomfort. Furthermore, there has been a scarcity of personal protective equipment to fight COVID-19, and this situation entails an increased probability of suffering different mental health consequences.

In this study, female midwives were more likely to develop depression as compared with men. This result was in line with the study done in China and Turkey (20, 21), this could be due to the intersect effect of genetic, biological, hormonal, social, and psychological factors. The second reason could be that the presence of androgen receptors in men may give protection, and since testosterone hormone does not cycle as estrogen in women, it also has protection in men. Because of the above reasons, women experience a high prevalence of mood and anxiety disorders (22). Moreover, in the Ethiopian context, women are responsible to take care of family and control their household activities in addition to their professional work, which makes them anxious and more stressed.

Those midwives working in rural areas were more likely to develop depression as compared with their counterparts. This finding is supported by a study done in China (23). This might be due to a lack of personal protective equipment in health facilities or poor knowledge and practice of the rural community toward COVID-19, which put midwives at an increased risk of developing depression.

Unlike depression, those midwives working in rural areas are less likely to develop stress compared with their counterparts. The possible reason might be that the burden of infection is highly prevalent in the urban area. Another possible reason could be isolation, quarantine, and treatment centers for COVID-19 are located in urban areas, so health professionals, such as midwives, who are working in urban health facilities, are worried about the risk of acquiring the infection.

Our study showed that midwives working in government health institutions have a higher prevalence of depression and anxiety as compared with those midwives working in private ones. This finding is in line with a systematic and meta-analysis study (24). This could be due to a critical shortage of PPEs in government health facilities where they are under the continuous threat of acquiring the infection.

Midwives who had poor knowledge of COVID-19 were more likely to develop depression and stress as compared with their counterparts. This is in line with studies done in China (25–27). This might be due to that health providers with a good knowledge of the COVID-19 outbreak are crucial in reducing the rate of infection and protecting from infection (28), which helps to avoid adverse psychological distress. Additionally, having a good knowledge of prevention and method of transmission of the novel virus would help the healthcare providers in engaging mindfully in activities, such as hand washing, wearing a mask, and keeping physical distancing, which minimizes the level of depression and stress (29).

The poor preventive practice of COVID-19 among providers had a significant association with DASS. This is supported by a study done in Saudi Arabia (28). This is due to the fact that respondents who practiced preventive measures for COVID-19 are less likely to acquire the infection, so they are less likely to develop stress, anxiety, and depression symptoms (30). Moreover, this could be due to the fact that taking care of and complying with the disease prevention strategies could give a sense of protection, and as a frontline cadre to care for patients; good prevention practice may reduce frustration and worry related to acquiring and transmitting the disease which leads to develop good mental health and wellbeing.

Midwives who had a poor attitude toward COVID-19 were more likely to develop anxiety than their counterparts. This finding is supported by a review article done among nurses and healthcare professionals (31). This might be because those people who are not confident that the disease can finally be controlled and have a higher perception of susceptibility and severity may feel more anxious.

Furthermore, exposure to substances was significantly and positively associated with depression and stress. This finding is in agreement with the study done in India (32). According to the World Health Organization (WHO) recommendation, alcohol consumption can increase the risk of catching COVID-19 (33). This might be also due to the triggering effect of substance, which impairs the decision-making and judgment ability of workers and reduce the preventive measures for COVID-19 infection. Moreover, poor COVID-19 preventive practice resulted in worry and fear that might be putative factors for the impairment of mental health and wellbeing.

The study has some limitations that need to be acknowledged. The study did not include those midwives who had no mobile network access. As the study findings are based on self-report using a subjective scale, there might be reporting bias among study participants. Due to the cross-sectional nature of the study, it does not show the cause and effect relationship.

## Conclusion

We found high rates of negative mental health outcomes among midwifery professionals in Ethiopia. The findings of this study have paramount importance to the public and clinical setups, and the findings of the study highlights the need for addressing knowledge and preventive practice gaps through information, training, and safety protocols on the COVID-19 modes of transmission, and prevention-related precautions as well as the provision of adequate personal protective equipment is essential to preserve the mental health of midwives during the COVID-19 pandemic. Moreover, the relative contribution of COVID-19 on the psychological disorders among midwives needs to be evaluated using a longitudinal study design.

## Data availability statement

The original contributions presented in this study are included in the article/supplementary material, further inquiries can be directed to the corresponding author.

## Ethics statement

This study was approved by the Institutional Review Boards of Woldia University (Ref: WDU/986/RCS/2020). Informed consent was obtained from participants after providing all necessary information on the study, informing them all the purpose, benefit, risk, and confidentiality of the information and the voluntary nature of the participation in the study. Participants were informed that all data obtained from them were kept confidential and no identifying information was collected from them.

## Author contributions

EK: writing—original draft, data curation, investigation, methodology, and formal analysis. BK: data curation, investigation, methodology, formal analysis, and supervision. ST: data curation, investigation, and methodology. AB: funding acquisition, investigation, supervision, methodology, formal analysis, and writing—review and editing. All authors contributed to the article and approved the submitted version.

## Abbreviations

AOR: Adjusted Odds Ratio
COVID-19: Coronavirus Disease 2019
CI: Confidence Interval
COR: Crude Odds Ratio
MERS-CoV: Middle East Respiratory Syndrome
EMwA: Ethiopian Midwives Association
SARS: Sever Acute Respiratory Syndrome
WHO: World Health Organization.
